# Antimicrobial activity of endophytic bacterial populations isolated from medical plants of Iran

**Published:** 2017-02

**Authors:** Maryam Beiranvand, Mansour Amin, Abdolrazag Hashemi-Shahraki, Bizhan Romani, Sajad Yaghoubi, Parisa Sadeghi

**Affiliations:** 1 Health Research Institute, Infectious and Tropical Diseases Research Center, Department of Microbiology, School of Medicine, Ahvaz Jundishapur University of Medical Sciences, Ahvaz, Iran; 2 Department of Epidemiology, Pasteur Institute of Iran, Tehran, Iran; 3 Cellular and Molecular Research Center (CMRC), Faculty of Medicine, Ahvaz Jundishapur University of Medical Sciences (AJUMS), Ahvaz, Iran; 4 Department of Biochemistry, University of Alberta, Edmonton, Alberta, T6G 2E1, Canada; 5 Division of Microbiology, Department of Pathobiology, School of Public Health and Institute of Public Health Research, Tehran University of Medical Sciences, Tehran, Iran; 6 Clinical Biochemistry Research Center, Shahrekord University of Medical Sciences, Shahrekord, Iran

**Keywords:** Endophytic, *Actinomycets*, Medicinal plants, Antimicrobial

## Abstract

**Background and Objectives:**

Endophytic actinobacteria colonize inside the plant tissues without causing damages to the host plant. Since these microorganisms colonize in the different parts of plants and can stop plant disease, they have been applied as biological agents for controlling human diseases. The aim of this study was molecular identification and measuring the antimicrobial activity of endophytic *Actinomycete*s isolated from medicinal plants of Iran.

**Materials and Methods:**

The total of 23 medicinal plant samples were collected, sterilized, and crushed. Small pieces of the crushed samples were then cultured directly on three selective media. Grown colonies were identified by 16S rRNA gene sequencing method. Each isolate was cultured in TSB medium and then antimicrobial compound was extracted using ethyl acetate and tested against the target bacteria.

**Results:**

Sixteen out of 23 bacterial isolates (69%) exhibited antimicrobial activity against the selected pathogenic bacteria, such as *Bacillus cereus, Staphylococcus aureus, Bacillus subtilis, Klebsiella pneumoniae, Citrobacter freundii, Proteus mirabilis, Shigella flexneri* and *Escherichia coli.*

**Conclusion:**

Our Study showed a high phylogenetic diversity and the potent antibiotic activity of endophytic bacteria in medicinal plants of Iran.

## INTRODUCTION

Natural sources of drugs have played important roles in medicine during the last decades. Since 1981 to 2006 nearly 70% of new drugs and chemical agents had natural sources (1). Over 22,000 natural products are isolated from microorganisms. Actinobacteria alone produce more than 45% of antibiotics in the world. Actinobacteria are Gram-positive bacteria with high guanine and cytosine content in their DNA. Some of them form filaments that resemble mycelia of the unrelated fungi (2, 3). Actinobacteria are frequently found in soil microflora and produce bioactive compounds including antibiotics (4, 5), antitumor compounds (6, 7), enzymes (8), and immunosuppressive agents (9). Due to the increasing emergence of bacterial resistance and replacing potent antimicrobial medicines, it is very important to focus on new antimicrobial sources. Actinomycete bioactive compounds are used safely in human and veterinary medicine products (3, 9). Many actinobacteria enter the inner tissues of plants and act either as pathogen or endophytic (6). The actinomycetes that reside in the inner tissues of living plants are known as endophytic actinobacteria (10, 11). They live in roots, stems, flowers, fruits, seeds or in many other tissues of plants (12) where they can stimulate growth of the host plants under adverse conditions and also fight plant diseases (13). Many of metabolites produced by these bacteria have antibacterial activities such as munumbicins A–D (13–15), celastramycins A–B (16), kakadumycins (17), and dimethyl novobiocins (18), which are isolated from *Sterptomyces spp.* (19). The aim of this study was to isolate and identify antimicrobial activity of endophytic actinomycets in medicinal plants.

## MATERIALS AND METHODS

### Sample collection.

Twenty three plant samples, including leaves, flowers, and fruits, were collected from four different provinces of Iran (Khuzestan, Tehran, Khoramabad, and Ilam) between 2013 and 2104. Plants were identified by Department of Botany at Chamran University, Iran. For bacterial isolation, each part of the plants was placed into a sterile plastic bag and transferred to the microbiology laboratory of Ahvaz Jundishapur University of Medical Sciences.

### Surface sterilization.

The collected samples were washed by tap water, dried, and processed by a five step surface sterilization procedure, which included 4–10 min washing with 5% NaOCl, 10 min wash with 2.5% Na_2_S_2_O_3_, 5 min wash with 75% ethanol, one wash with sterile water, and final rinse with 10% NaS_2_O_3_ for 10 min. All samples were then dried at 100°C for 10 min (10). To check the sterilization, randomly surface-sterilized tissues were imprinted on blood agar (Merck Germany), incubated at 28°C for 2 days, and examined for microbial growth (10, 14).

### Isolation of endophytes.

**Fig. 1.**
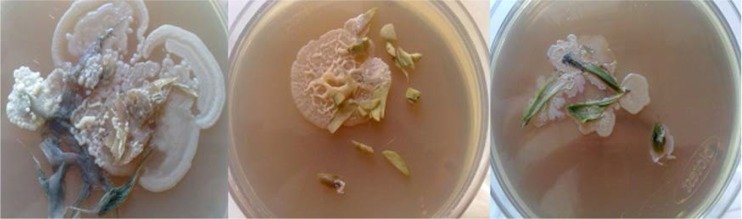
Colonies of endophytic bacteria grown on R3A culture medium

Three samples of each plant were aseptically crushed into small fragments and placed directly on the international streptomyces project 2 (ISP2) agar (HI Media, India), R3A agar (1g yeast extract, 1g protease-peptone, 1g casamino acid, 1g glucose, 1g starch, 0.5 g sodium pyruvate, 0.6 g K_2_HPO_4_, 1g MgSO_4_ 7H_2_O, and 18g agar in 1 liter distilled water), and blood agar. Plates were incubated at 28°C for 2 to 4 days. Bacterial isolates were identified by their morphology and characteristics of their colonies such as size, shape, colour, and growth rate at different temperatures (Fig. 1). Colonies with similar morphological features were grouped into the same species (15).

### Molecular identification.

16S rRNA gene was amplified using specific primer for selected isolates. The PCR products were sequenced using 27F (5′-AGAGTTTGATCCTGGCTCAG-3′), 1525-R (5′AAGGAGGTGWTCCARCC-3′) and 907-R (5′-CCGTCAATTCMTTTRAGTTT-3′) with an ABI 3100 genetic analyser. The PCR was carried out with an initial denaturation at 94°C for 2 min, 30 cycles of 94°C for 1 min, 60°C for 1 min, 72°C for 3 min, and final extension at 72°C for 10 min. Dye Terminator Cycle Sequencing Genome Lab™ was used to determine the sequence of PCR products according to the manufacturer’s instructions (Biometra Co, Germany).

### Phylogenetic analysis.

Multiple alignments of the 16S rRNA were generated using sequences of our bacterial isolates and the bacterial strains from Gen-Bank database with the jPhydit program (16). MEGA version 4 (17) was used to construct a neighbour-joining phylogenetic tree using the Kimura two-parameter. The reliability of the branching and clustering pattern was estimated from 1000 bootstrap replicates. The tree was rooted with *E. coli* 16S rRNA.

### Extraction of antimicrobial compounds.

The isolates were cultured in TSB (Tryptic Soy Broth, Merck Germany) and incubated at 28°C for 14 to 16 days, after which we proceeded with extraction of antimicrobial compounds using 3 methods. In the first method, one part of the cultured media was mixed with one part ethyl acetate (1:1 ration) and stirred with magnetic stirrer for 6 h. The organic supernatant was separated and centrifuged at 5,000 rpm for 10 min. The ethyl acetate layer was transferred into a clean flask, and dried with rota-evaporator (Heidolph, Germany) at 50°C. The dry extract was dissolved in 2 ml of ethanol. To test antimicrobial susceptibility of the extracts, 25 μl of each extract in ethanol was used to soak blank discs (7 mm in diameters) (18). In the second method, another part of media was incubated in boiling water for 5 min and in cold water for 5 min then added one part of cultured media were mixed with one part ethyl acetate (1:1 ratio). As described for the first method, samples were then processed. In the third method, the last part of media, which contained bacteria, was sonicated for 3 min at 160W and extraction method was completed as described for the first method. Bacterial isolates obtained using the 3 methods were tested by disc diffusion against pathogenic bacteria, including *Staphylococcus aurous, Bacillus subtilis, Pseudomonas aeruginosa, Citrobacter freundii, Shigella flexneri, Escherichia coli, Klebsiella pneumoniae, Bacillus cereus,* and *Proteus mirabilis*. The inhibition zone was measured for each bacterial species separately.

## RESULTS

### Antimicrobial susceptibility.

**Fig. 2.**
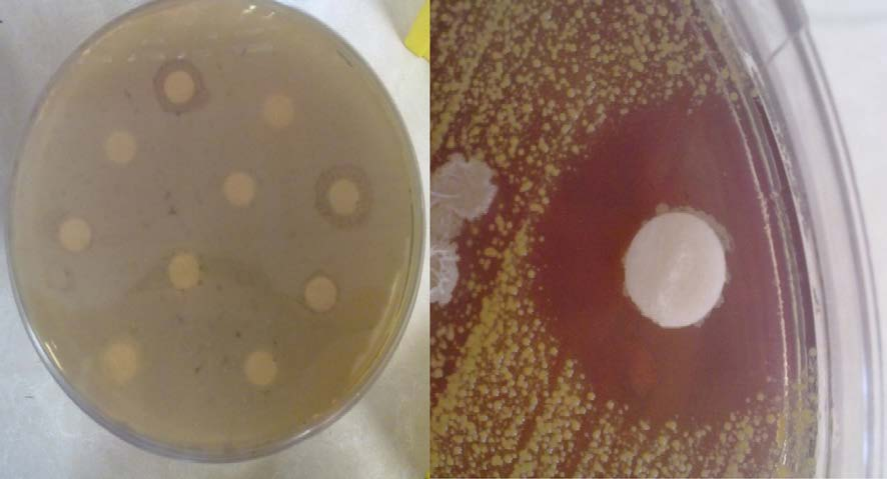
Antimicrobial susceptibility test of endophytic bacteria

Using the plant tissues crushed and cultured in Petri dishes, plant-associated bacteria were successfully isolated (Fig. 1). The antibacterial activity of the isolated bacteria was examined using the antibiotic disc diffusion method. All isolated bacteria displayed anti-bacterial activities against the selected bacteria for antibiotic susceptibility. Among the isolated bacteria, 16 isolates (69.56%) obtained by the second method (hot method) showed strong antiviral activities by producing large inhibition zones. Using the third method (ultrasonic method) 13 isolates (56.52%) were obtained that all displayed antiviral activities. Among the isolates of the third method, EB7 (isolated from the root of *Stachys lavandulifolia*) and EB69 (isolated from the root of *Physalis alkekengi*) displayed a broad antibacterial activity against all the target bacteria (Table 1, Fig. 2).

**Table 1.** Antimicrobial activity^*^ of endophytic bacteria extracted by 2 methods against pathogenic bacteria

	Antimicrobial compounds extracted with hot method								Antimicrobial compounds extracted with ultrasonic method							
	
**Isolate number**	***B. cereus***	***S. aureus***	***B. subtilis***	***K. pneumoniae***	***C. freundii***	***P. mirabilis***	***Sh. Flexneri***	***E. coli***	***B. cereus***	***S. aureus***	***B. subtilis***	***K.pneumoniae***	***C. freundii***	***P. mirabilis***	***Sh. flexneri***	***E. coli***
EB4	+++	−	−	−	−	−	−	−	−	−	−	−	−	−	++	−
EB5	+++	+++	+++	−	++	−	+	−	−	−	−	−	+++	−	−	−
EB14	−	−	−	++	++	−	++	−	−	−	−	++	++	−	++	−
EB18	−	−	+++	+	−	+	+++	−	−	−	−	+++	−	−	−	−
EB22	−	−	−	−	−	−	+	+	−	−	−	++	+++	−	+++	−
EB23	+++	−	−	+	−	−	+	−	−	−	−	−	+++	−	−	−
EB25	+	+	++	+	−	−	+	−	−	−	−	+++	−	−	++	−
EB26	+++	−	+++	+	−	−	+	−	−	−	−	++	−	−	++	−
EB97	−	−	−	−	−	−	−	−	−	−	+++	−	+++	−	−	−
EB 133	+	−	+	−	−	+	−	+	−	−	−	−	++	−	−	−
EB3	−	++	−	−	−	−	−	−	−	−	−	−	−	−	−	−
EB 6	−	−	−	−	−	−	−	−	−	−	−	−	−	−	−	−
EB 7	+++	+++	+++	+++	+++	+++	+++	+++	−	−	+	+++	+++	−	+++	
EB 8	−	−	−	−	−	−	−	−	−	−	−	−	−	−	−	−
EB9	−	−	−	−	−	−	+	−	−	−	−	−	−	−	−	−
EB 10	+	−	−	−	−	−	−	−	−	−	−	−	−	−	−	−
EB11	−	−	−	−	−	−	−	−	−	−	−	−	−	−	−	−
EB12	−	−	−	−	−	−	−	−	−	−	−	−	−	−	−	−
EB17	−	−	++	−	−	−	++	−	−	−	+++	++	−	−	+++	−
EB 19	−	−	−	−		−	−	−	−	−	−	−		−	−	−
EB20	−	−	−	−	−	−	−	−	−	−	−	−	−	−	−	−
EB64	+	+	−	−	−	−	−	−	−	−	−	−	−	−	−	−
EB69	+++	+++	+++	+++	+++	+++	+++	+++	−	−	−	+++	+++	−	++	−

* Inhibition zone, EB: Endophytic Beiranvand (Beiranvand is surname of the first author), − : No activity, +: weak activity, ++: moderate activity, +++: high activity

### Identification of bacteria.

**Fig. 3.**
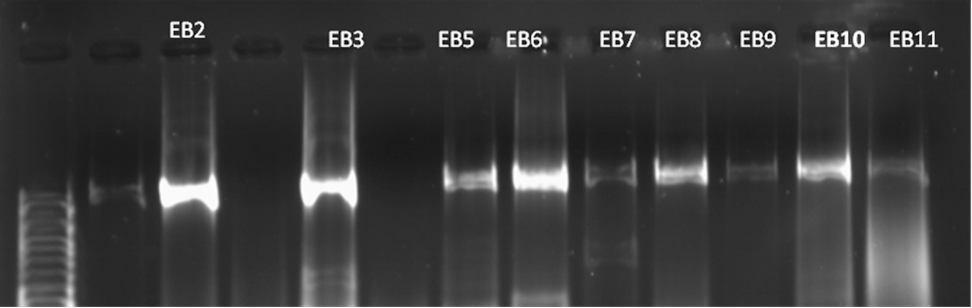
Identification of endophytic bacteria using 16S rRNA

We sequenced and performed phylogenetic analysis of 23 bacterial isolates obtained from 23 plants. The results of phylogenetic analysis of 16S rRNA (Table 2 and Fig. 3) showed that our isolates belonged to different bacterial species, including Gram negative (such as *Pseudomonas graminis*) and Gram positive bacteria (such as *Bacillus thuringiensis*). Among our isolates, there were also three isolates whose species could not be identified. These isolates, whose genera were identified, included isolate EB4 (*Planomicrobium* Sp), EB11 (*Bacillus* sp), EB12 (*Staphylococcus* Sp), and EB23 (*Bacillus* Sp). Taken together, our results suggested that there is wide range of diversity among endophytic bacteria.

**Table 2.** Taxonomic characterisation of actinobacteria isolated from different plant organs.

**Isolate**	**Host plant**	**Sampling Location**	**Organ**	**Species**	**Genbank accession**	**similarity**
EB 3	*Allium schoenoprasum*	Khorramabad	Root	*Bacillus aryabhattai*	*KP209388*	99/44
EB 4	*Mentha pulegium*	Ahvaz, Mullah Agha	Root	*Planomicrobium Sp*	*KP324949*	99/34
EB 5	*Marrubium vulgare*	Ahvaz, Salnd Mountain	Root	*Actinoallomuru acacia*	*KP209377*	100
EB 6	*Falcaria vulgaris*	Khorramabad	Root	*Actinoallomurus oryzae*	*KP209378*	100
EB 7	*Stachys lavandulifolia*	Tehran, Damavand	Root	*Amycolatopsis tolypophora*	*KP209379*	100
EB 8	*Ocimum basilicum*	Ahvaz	Root	*Bacillus polyfermenticus*	*KP209385*	100
EB 9	*Alcea amcheri*	Ahvaz, Salnd Mountain	Stem	*Dietzia cercidiphylli*	*KP209389*	99/29
EB 10	*Chenopodium album*	Khorramabad	Root	*Bacillus pumilus*	*KP209384*	99/29
EB 11	*Gundelia tournefortii*	Khorramabad	Rood	*Bacillus. Sp*	*KP324948*	100
EB 12	*Achillea millefolium*	Ahvaz, Imam Abdullah	Root	*Staphylococcus Sp*	*KP324950*	99/93
EB14	*Borago officinalis*	Ahvaz	Leaf	*Blackwater bioreactor*	*KP209383*	98/96
EB 17	*Allium ursinum*	Tehran, Damavand	Stem	*Azorhizobium caulinodans*	*KP209380*	99/79
EB 18	*Zataria multiflora*	Tehran, Damavand	Root	*Azospirillum brasilense*	*KP209381*	98/55
EB19	*Chenopodium album*	Khorramabad	Root	*Bacillus velezensis*	*KP209386*	100
EB20	*Lavandula angustifolia*	Tehran, Damavand	Root	*Planomicrobium chinense*	*KP209393*	100
EB22	*Cymbopogon oliviery*	Ahvaz, Haftcal road	Rood	*Nocardia niigatensis*	*KP209393*	100
EB 23	*Phasaeolous vulgaris*	Khormabad	Stem	*Bacillus Sp*	*KP209390*	100
EB 25	*Teucrium polium*	Ahvaz, Salnd Mountain	Root	*Pseudomonas graminis*	*KP209394*	100
EB 26	*Aloe vera*	Khorramabad	Leaf	*Arthrobacter globiformis*	*KP209382*	100
EB64	*Cucumis sativus*	Khorramabad	Fruit	*Nocardia cyriacigeorgica*	*KP209396*	100
EB 69	*physalis alkekengi*	Ahvaz	Root	*Bacillus thuringiensis*	*KP209387*	100
EB97	*Rheum rhaponticum*	Tehran, Damavand	Stem	*Streptomyces artemisiae*	*KP209395*	100
EB 133	*Coriandrum sativum*	Khorramabad	Root	*Microbacterium testaceum*	*KP209391*	100

## DISCUSSION

Since actinobacteria have been applied in producing different kinds of current medicines, more recent studies are investigating new bacterial sources. This study focused on actinobacteria, in particular actinomycets, which was found in a large number of the isolated bacteria obtained from selected plants. We isolated 23 endophytic bacterial isolates, among which species of 19 isolates were identified by 16S rRNA. The majority of the isolates could be considered for producing antibiotics against target bacteria. Two out of 23 endophytic bacterial isolates, EB4 and EB7, showed inhibitory activity against *Bacillus cereus*. In addition, EB9 showed inhibitory activity against *Staphylococcus aureus, Citrobacter freundii* and *Shigella flexneri*. Sixteen isolates (69%) obtained by hot method showed strong activity against selected pathogenic organisms and two of them (EB7 and EB69) had broad spectrum antibacterial activity (Table 1). Ultrasonic method showed that 13 out of 23 isolates (46%) inhibited microbial growth. Studies have shown that most of the isolated organisms were *Bacillus* (25, 26). Similarly to our study, Janso et al. reported that 105 out of 123 endophytic actinomycets isolated from tropical plants belonged to 17 different genera and also *Sphaerisporangium* and *Planotetraspora* as rare genera, which have not been reported previously. They had nearly 60% bioactive activities (25). Strobel et al. isolated *Streptomyces, Microbispora,* and *Nocardiodes* as endophytic bacteria and showed that their isolates produce antimicrobial compounds with inhibitory effects against Gram positive bacteria (11). Another study isolated 560 endophytic actinomycetes from 26 herbal species. Their isolates, which belonged to a wide range of bacterial species, showed strong antimicrobial properties (27).

**Fig. 4.**
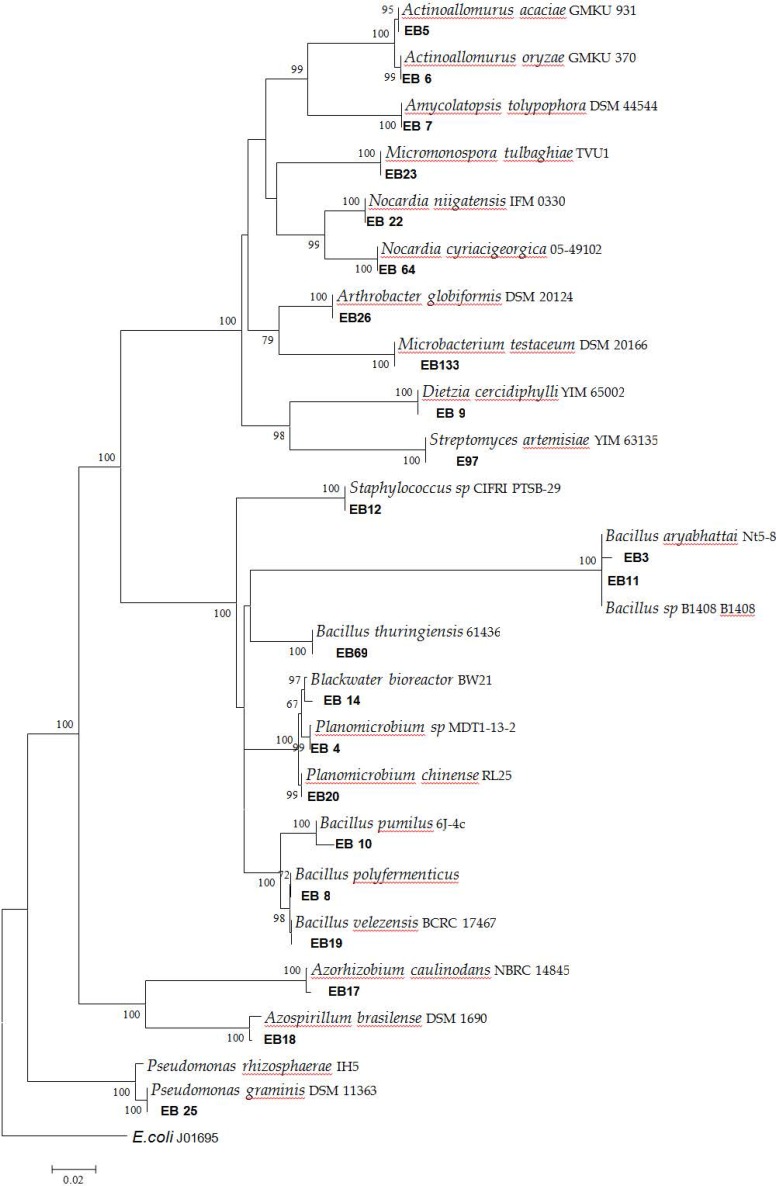
Phylogenetic tree constructed using 16s rRNA gene sequences.

## CONCLUSION

Overall, study revealed a high phylogenetic diversity among endophytic bacteria isolated from different areas of Iran. Our isolates showed considerable antimicrobial activities. This study demonstrated that hot method was more efficient than the other method for isolating endophytic bacteria. Endophytic actinobacteria can be used for producing new bioactive compounds. Such compounds can be used against pathogenic bacteria resistant to the current antibiotics.
